# Breast radiation dose with contrast-enhanced mammography-guided biopsy: a retrospective comparison with stereotactic and tomosynthesis guidance

**DOI:** 10.1007/s00330-024-10920-3

**Published:** 2024-08-14

**Authors:** Rodrigo Alcantara, Javier Azcona, Mireia Pitarch, Natalia Arenas, Xavier Castells, Pablo Milioni, Valentina Iotti, Giulia Besutti

**Affiliations:** 1 Department of Medicine, Universitat Autònoma de Barcelona, Barcelona, Spain; 2 Radiology and Nuclear Medicine Department, Hospital del Mar, Parc de Salut Mar, Barcelona, Spain; 3 Epidemiology and Evaluation Department, Hospital del Mar Research Institute (IMIM), Barcelona, Spain; 4 Health Services Research on Chronic Patients Network (REDISSEC), Institute of Health Carlos III, Madrid, Spain; 5 GE Healthcare, Buc, France; 6 Radiology Unit, Department of Diagnostic Imaging and Laboratory Medicine, Azienda USL-IRCCS di Reggio Emilia, Reggio Emilia, Italy; 7 Department of Medical and Surgical Sciences, University of Modena and Reggio Emilia, Modena, Italy

**Keywords:** Breast neoplasms, Mammography, Contrast media, Biopsy, Radiation dosage

## Abstract

**Objectives:**

This retrospective study aimed to compare the average glandular dose (AGD) per acquisition in breast biopsies guided by contrast-enhanced mammography (CEM), conventional stereotactic breast biopsy (SBB), and digital breast tomosynthesis (DBT). The study also investigated the influence of compressed breast thickness (CBT) and density on AGD. Furthermore, the study aimed to estimate the AGD per procedure for each guidance modality.

**Methods:**

The study included 163 female patients (mean age 57 ± 10 years) who underwent mammography-guided biopsies using SBB (9%), DBT (65%), or CEM (26%) guidance. AGD and CBT data were extracted from DICOM headers, and breast density was visually assessed. Statistical analyses included two-sample *t*-tests and descriptive statistics.

**Results:**

Mean AGD per acquisition varied slightly among CEM (1.48 ± 0.22 mGy), SBB (1.49 ± 0.40 mGy), and DBT (1.55 ± 0.47 mGy), with CEM presenting higher AGD at lower CBTs and less dose escalation at higher CBTs. For CBT > 55 mm, CEM showed reduced AGD compared to SBB and DBT (*p* < 0.001). Breast density had minimal impact on AGD, except for category A. The estimated AGD per procedure was approximately 11.84 mGy for CEM, 11.92 mGy for SBB, and 6.2 mGy for DBT.

**Conclusion:**

The study found mean AGD per acquisition to be similar for CEM and SBB, with DBT slightly higher. CEM demonstrated higher AGD at lower CBT but lower AGD at higher CBT, indicating reduced dose escalation with increasing thickness. While breast density had minimal overall impact, variations were noted in category A. DBT was more dose-efficient per procedure due to fewer acquisitions required.

**Clinical relevance statement:**

CEM guidance provides effective lesion visualization within safe radiation limits, improving the precision of percutaneous image-guided breast interventions and supporting its potential consideration in a wider range of breast diagnostic procedures.

**Key Points:**

- *Limited data exist on the AGD using CEM guidance for breast biopsies*.
- *CEM and SBB exhibit similar AGD per acquisition; DBT demonstrated the lowest AGD per procedure*.
- *Radiation from CEM guidance fits within safe limits for percutaneous image-guided breast interventions*.

**Graphical Abstract:**

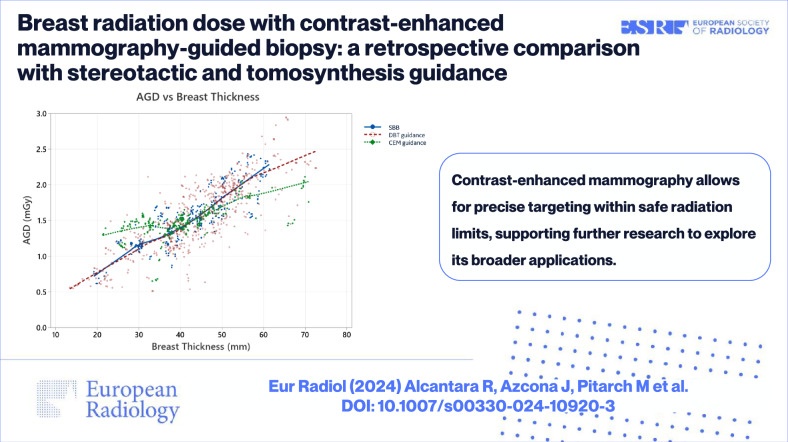

## Introduction

Contrast-enhanced mammography (CEM) is an emerging imaging technique based on the acquisition of a dual-energy mammogram, which is acquired approximately two minutes after the intravenous injection of iodinated contrast media [1]. CEM consists of a low-energy (LE) image that resembles a conventional 2D mammogram, providing morphologic details, and a recombined image (RC) that displays the distribution of the contrast media, revealing changes in breast perfusion often associated with neoplastic angiogenesis.

Suspicious non-palpable findings typically require histological evaluation, usually performed using breast imaging guidance through techniques such as core-needle or vacuum-assisted biopsies. CEM guidance introduces a novel approach in breast interventional radiology, which employs dual-energy stereotactic acquisitions post-iodinated contrast media administration, enhancing the visualization of suspicious abnormalities. Initial reports confirm the technical feasibility and high success rate of CEM-guided biopsies [2, 3].

Alternative contemporary mammographic methods employed for percutaneous breast interventions include conventional stereotactic breast biopsy (SBB) and digital breast tomosynthesis (DBT) [4, 5]. While both SBB and CEM-guided procedures typically require a minimum of three acquisitions to determine the target coordinates, DBT-guided interventions usually necessitate only a single acquisition [5].

The average glandular dose (AGD), typically measured in milligrays (mGy), is the key metric for evaluating exposure doses in mammographic techniques, reflecting the dose absorbed by mammary tissue [6]. This metric, which can be estimated from technical parameters using the method proposed by Dance et al [7–9], is widely recognized in medical imaging and radiotherapy [9–11]. Lower AGD values are generally associated with a reduced risk of radiation-induced breast cancer [10, 11].

Comparative studies have analyzed AGD across various mammographic techniques. Routine DBT shows a slight AGD increase over digital mammography (DM) [12, 13], while CEM often exhibits higher radiation levels than DM but may be comparable to or less than DBT [14–16]. These differences are associated with factors like breast thickness and density, which correlate with rising AGD [17]. However, such studies typically focus on routine diagnostics, with less emphasis on interventional applications. Early reports on CEM-guided biopsy have revealed a gap in detailed AGD data [2, 3, 18], with recent publications beginning to fill this void, though comprehensive data are still needed [19, 20].

The primary aim of this study was to compare the AGD obtained using CEM guidance with that from other mammography-guided techniques, such as SBB or DBT, exploring its relationship with compressed breast thickness (CBT) and density. We analyzed the AGD for each image acquisition and extended our evaluation to estimate AGD per procedure, based on the typical number of acquisitions by each technique according to our institution’s protocol. Additionally, we assessed the contributions of LE and high-energy (HE) images to the overall radiation dose in CEM guidance.

## Methods

### Study design and ethics

This retrospective study compared the AGD in patients undergoing breast biopsies through different mammography-guided techniques, including SBB, DBT-guided, and CEM-guided procedures. Informed consent was secured for all procedures, with additional consent for contrast administration in CEM. Patients who underwent CEM-guided biopsy were part of a prospective study [2], approved by the hospital ethics committee (protocol number 2019/8890), for which they provided additional written informed consent for research. For retrospective analyses involving SBB and DBT-guided biopsies, ethical approval was obtained, and the need for additional consent was appropriately waived.

### Study population

The study included all consecutive patients who underwent mammography-guided procedures between June 2018 and September 2021, using the same mammographic equipment, specifically the Senographe Pristina™ (GE Healthcare), equipped with automatic optimization of parameters (AOP). Only cases with a complete image dataset available were included. We excluded any cases presenting incomplete datasets, as well as those acquisitions involving spot-magnified views or specimen images, to maintain the data’s integrity and reliability in our analysis.

### Interventional mammographic guidance techniques

SBB and CEM-guided procedures commence with the acquisition of a scout image, which is captured perpendicular to the image detector to provide an initial overview of the lesion. After this, additional images are acquired at 15-degree intervals, both positive and negative relative to the original scout image plane, to ascertain the three-dimensional coordinates of the lesion, which are essential for precise targeting. After injecting local anesthesia, and especially in cases of subtle findings, we may need to take extra images to compensate for any shifts caused by the anesthesia itself. This check is repeated after inserting the biopsy needle, in a pre-fire mode, to confirm its correct alignment with the lesion. A final image after the biopsy verifies both the successful retrieval of the lesion and the accurate placement of the clip marker. Our prior research on CEM-guided biopsies indicated that, typically, an average of two scout images are required for precise initial alignment [2]. According to our standard protocol, at least eight images are needed per procedure, as shown in Supplementary Fig. 1.

DBT-guided procedures, however, adopt a different strategy by using a single multiplanar scout image to directly estimate the lesion’s depth (*z*-coordinate), thereby reducing the need for multiple image pairs. This approach usually involves about four images, potentially resulting in a lower overall AGD due to fewer steps and acquisitions [21]. Nonetheless, the actual number of images taken can vary with each modality, influenced by factors like the initial clarity of the lesion and any necessary adjustments for patient movement or lesion shift.

### Equipment and AGD assessment

Interventional acquisitions for SBB, DBT, and CEM in this study were performed using the same equipment—the Senographe Pristina™, along with the Serena™ and Serena Bright™ addons (GE HealthCare), all operating under AOP mode. The AOP dynamically adjusts technical factors such as kVp and mA based on the detected properties of breast tissue during an initial scout-view exposure. For instance, the AOP uses lower settings for polymethylmethacrylate (PMMA) phantoms representing breasts with a CBT under 40 mm, while it increases settings for phantoms over 50 mm to effectively image thicker tissues [22, 23]. Notably, for SBB and DBT-guided interventions, the only AOP setting available is an upscaled dose level designated as standard plus (STD +) mode, distinct from the standard (STD) mode used for routine and interventional CEM, routine DM, and routine DBT. Moreover, kVp and filtration settings remain consistent across SBB and DBT modalities. This tailored approach ensures that each modality receives an optimized radiation dose, adjusting for factors such as breast composition and thickness, thus accommodating procedural needs and variations in patient anatomy.

### Data collection

Metadata was retrieved from the anonymized DICOM headers of all images acquired during the interventional procedures and transferred to a structured spreadsheet. This dataset encompassed key parameters, including AGD and CBT, facilitating a nuanced analysis of the radiographic exposure and imaging conditions.

Breast density was determined visually in accordance with the breast composition categories of the American College of Radiology (ACR) BI-RADS® mammography lexicon [24], employing routine images corresponding to each interventional case for consistency. In the context of CEM-guided interventions, the low-energy image (LE) was used for density assessment, due to its analogous characteristics to conventional mammography [25]. The evaluations were carried out by a dedicated breast radiologist with 10 years of experience in mammographic imaging and breast density assessment (R.A.).

### Statistical analysis

The extracted data were organized according to the biopsy guidance modality used: SBB, DBT, and CEM. Data obtained from each acquisition facilitated a comparative evaluation of the AGD per single acquisition across different modalities. AGD has also been compared per procedure by considering the estimated mean number of acquisitions for each modality. Additionally, AGD was evaluated as a function of breast thickness and within various breast density categories, comparing different guidance modalities. CBT was classified in 5 mm intervals (e.g. the 20 mm category includes thicknesses from 15 mm to 25 mm) and further divided into ‘thin’ (< 35 mm), ‘thick’ (> 55 mm), and ‘mid’ (all other measurements). This categorization was applied to assess the influence of breast thickness on AGD. The AGD repartition between LE and HE exposures was also assessed in the subgroup of patients who underwent CEM guidance.

Statistical analysis was carried out using Minitab v20.3 (Minitab, Inc. 2020. http://www.minitab.com), a commercial computer software. Two-sample Student’s *t*-tests were employed to compare AGD across different biopsy guidance techniques, with an alpha set at 0.05 for statistical significance. This choice was predicated on the data’s suitability for parametric testing, given the assumption of normal distribution for AGD values and the aim to assess mean differences between modalities. Grouping SBB and DBT together increased the statistical power, and facilitated a direct comparison against CEM, performing the test between two populations, reflecting our interest in evaluating the novel CEM’s performance relative to established techniques. The analysis was conducted on a per-acquisition basis to standardize the comparison and mitigate the impact of procedural variability, thereby providing a more direct assessment of the inherent radiation dose characteristics of each guidance modality.

## Results

### Included patients

**Fig. 1:**
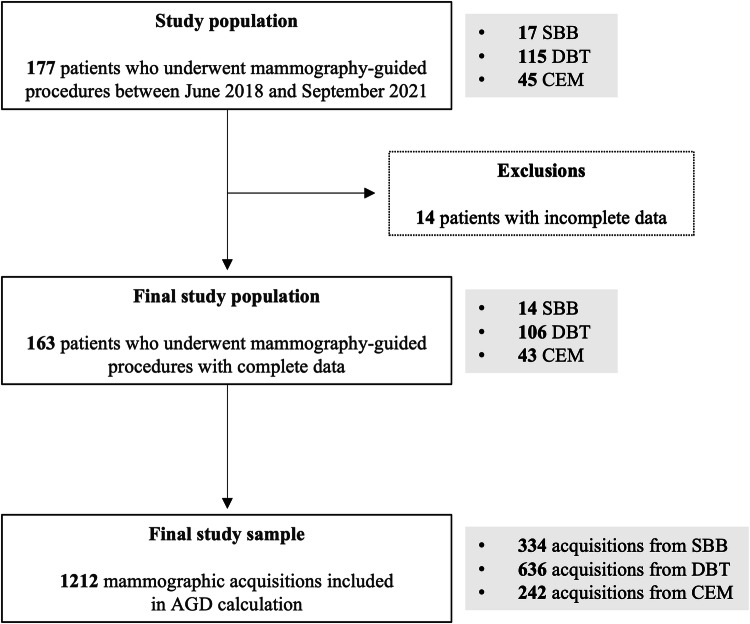
Flowchart of patient enrolment and acquisitions per guidance modality. SBB, stereotactic breast biopsy*;* DBT, digital breast tomosynthesis-guided biopsy; CEM, contrast-enhanced mammography-guided biopsy

Between June 2018 and September 2021, 177 patients underwent mammography-guided procedures. After excluding 14 patients with incomplete data, the study comprised 163 patients, accounting for 163 biopsies and 1212 mammographic acquisitions. A flowchart outlining the patient and procedure selection is provided in Fig. 1.

Table 1 summarizes the information on the included population. The average patient age was 57 years old and varied little with different guidance techniques (*p* = 0.292). The overall average CBT for the studied population was 42 mm. While the average CBT for SBB and CEM-guided procedures was consistent with this average, DBT-guided procedures had a slightly but significantly higher average CBT (*p* < 0.001).

**Table 1:** Patient demographics and procedure details by interventional guidance technique

	SBB	DBT	CEM	Total
Number of procedures* (Jun 2018–Sept 2021)	17 (10%)	115 (65%)	45 (25%)	177 (100%)
Procedures included in AGD calculations	14 (9%)	106 (65%)	43 (26%)	163 (100%)
Number of acquisitions*	334 (28%)	636 (52%)	242 (20%)	1212 (100%)
Patient age (years)^†^	59 ± 8	56 ± 9	59 ± 12	57 ± 10
95% CI for the mean age	(54.38, 63.75)	(54.49, 62.47)	(55.16, 62.47)	–
Breast density**				
A	3 (19%)	9 (8%)	–	12 (7%)
B	5 (31%)	53 (47%)	20 (44%)	78 (45%)
C	7 (44%)	45 (40%)	22 (49%)	74 (43%)
D	1 (6%)	5 (4%)	3 (7%)	9 (5%)
CBT (mm)^†^	41 ± 10	44 ± 11	41 ± 10	42 ± 11
95% CI for the mean for CBT	(39.96, 42.17)	(42.84, 44.58)	(39.31, 41.83)	–
CBT vs breast density^†^				
A	52 ± 8	55 ± 10	–	54 ± 9
B	44 ± 7	44 ± 10	42 ± 9	44 ± 9
C	37 ± 10	43 ± 12	40 ± 11	40 ± 11
D	39 ± 9	37 ± 10	36 ± 7	37 ± 9

*CI* confidence interval, *CBT* compressed breast thickness

^*^ Numbers of procedures and acquisitions are presented as count (percentage of total)

^**^ Numbers of procedures per density category are presented as count (percentage of total per technique)

^†^ Numerical values are presented as average ± 1 standard deviation (Std. Dev)

Most patients had breast density classified as either ACR category B or C for all modalities; SBB showed an increased proportion of patients with ACR category A compared to other modalities, while there was no ACR category A in the CEM guidance cohort. In our study, CEM guidance was typically reserved for RC-only findings, which are less commonly observed in category A breasts, known for their less dense tissue. Additionally, category A breasts had a higher mean CBT compared to other categories.

### AGD comparisons

Tables 2–4 provide a descriptive analysis of AGD, overall and as a function of breast thickness (binned at 10 mm steps) and density. The overall mean AGD for a single CEM-biopsy acquisition was 1.48 ± 0.22 mGy, which aligns closely with SBB (1.49 ± 0.40 mGy) and DBT (1.55 ± 0.47 mGy) guidance. The marginally higher AGD noted in DBT acquisitions likely stems from the increased CBT observed in this group, as outlined in Table 1.

**Table 2:** AGD measurements analysis across intervention guidance modalities

	AGD, (mGy)						
Modality	*N*	Avg	Median	Std. Dev	Min	Max	95% CI for avg
SBB	334	1.49	1.48	0.40	0.62	2.43	1.45–1.53
DBT	636	1.55	1.51	0.47	0.51	2.95	1.52–1.59
CEM	242	1.48	1.47	0.22	0.65	2.11	1.48–1.50

AGD is presented in mGy with the sample size (*N*), average (Avg), standard deviation (Std. Dev), minimum (Min), and maximum (Max) values for each modality

*SBB* stereotactic breast biopsy, *DBT* digital breast tomosynthesis, *CEM* contrast-enhanced mammography, *CI* confidence interval

**Table 3:** AGD measurements variation by CBT categories across intervention guidance modality

		AGD, (mGy)						
CBT, (mm)	Modality	*N*	Avg	Median	Std. Dev	Min	Max	95% CI for avg
10	SBB	–	–	–	–	–	–	–
	DBT	2	0.56	–	0.01	0.55	0.56	–
	CEM	–	–	–	–	–	–	–
20	SBB	14	0.77	0.72	0.19	0.62	1.21	0.66–0.88
	DBT	44	0.77	0.69	0.2	0.56	1.49	0.71–0.83
	CEM	3	1.33	1.31	0.05	1.3	1.39	–
30	SBB	86	1.19	1.15	0.16	0.96	1.7	1.16–1.23
	DBT	66	1.13	1.15	0.25	0.51	2.11	1.07–1.20
	CEM	69	1.34	1.42	0.23	0.65	1.6	1.29–1.40
40	SBB	119	1.4	1.45	0.22	0.84	1.91	1.36–1.44
	DBT	229	1.39	1.35	0.28	0.54	2.13	1.35–1.42
	CEM	105	1.42	1.43	0.11	0.95	1.65	1.40–1.45
50	SBB	78	1.79	1.75	0.25	1.26	2.32	1.74–1.85
	DBT	209	1.8	1.87	0.27	0.64	2.3	1.76–1.83
	CEM	45	1.65	1.63	0.14	1.3	2.05	1.61–1.69
60	SBB	37	2.1	2.16	0.3	1.54	2.43	2.00–2.19
	DBT	66	2.1	2.12	0.34	1.31	2.84	2.02–2.19
	CEM	9	1.84	1.83	0.14	1.53	2.03	1.73–1.94
70	SBB	–	–	–	–	–	–	–
	DBT	20	2.3	2.31	0.41	1.45	2.95	2.11–2.50
	CEM	11	1.79	1.95	0.28	1.43	2.11	1.61–1.98

Breast thickness categories are defined as intervals of ± 5 mm from the displayed value (e.g. the 20 mm category includes the range of 15–25 mm CBT)

*CBT* compressed breast thickness, *CI* confidence interval

**Table 4:** AGD measurements variation by ACR breast density categories across interventional guidance modalities

		AGD, (mGy)						
Breast density*	Modality	*N*	Avg	Median	Std. Dev	Min	Max	95% CI for avg
A	SBB	26	1.64	1.59	0.36	1.00	2.22	1.50–1.78
	DBT	39	1.90	1.93	0.63	0.70	2.95	1.70–2.11
	CEM	–	–		–	–	–	–
	All	65	1.80	1.73	0.55	0.70	2.95	1.66–1.93
B	SBB	124	1.51	1.48	0.35	0.84	2.33	1.44–1.57
	DBT	299	1.46	1.36	0.46	0.54	2.84	1.41–1.51
	CEM	95	1.49	1.44	0.23	0.94	2.11	1.44–1.54
	All	518	1.48	1.43	0.40	0.54	2.84	1.44–1.51
C	SBB	159	1.43	1.24	0.45	0.62	2.43	1.36–1.50
	DBT	269	1.61	1.67	0.44	0.51	2.42	1.56–1.67
	CEM	132	1.46	1.47	0.23	0.65	1.91	1.42–1.50
	All	560	1.53	1.49	0.41	0.51	2.43	1.49–1.56
D	SBB	25	1.58	1.68	0.22	1.18	1.78	1.49–1.67
	DBT	29	1.47	1.38	0.30	0.97	2.11	1.35–1.58
	CEM	15	1.49	1.49	0.08	1.35	1.65	1.44–1.54
	All	69	1.51	1.57	0.24	0.97	2.11	1.45–1.57

*CI* confidence interval

^*^ Density categories based on ACR BI-RADS®: A (almost entirely fatty), B (scattered fibroglandular densities), C (heterogeneously dense), and D (extremely dense)

**Fig. 2:**
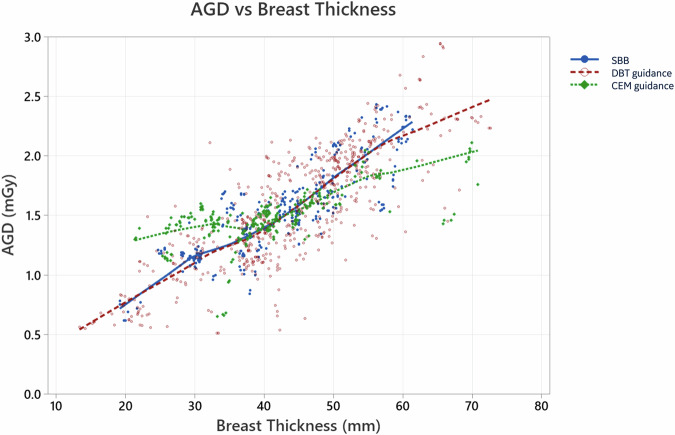
Scatter plot of AGD, in mGy, against breast thickness. Data points for each biopsy guidance modality are shown, with trend lines representing the moving average, indicating the relationship between AGD and breast thickness

For all guidance modalities, the AGD increased with increasing breast thickness, though this increase was less pronounced for CEM-guidance (Table 3 and Fig. 2). CEM demonstrated a slightly higher mean AGD in ‘thin’ breasts (CBT below 35 mm): 1.34 mGy (95% CI = 1.29–1.40) compared to 1.05 mGy (95% CI = 1.02–1.09) for the combination SBB + DBT (*p* < 0.001). Conversely, in ‘thick’ breasts (CBT above 55 mm), CEM showed a slightly lower mean AGD: 1.81 mGy (95% CI = 1.71–1.92) versus 2.13 mGy (95% CI = 2.07–2.20) for the combination SBB + DBT (*p* < 0.001).

**Fig. 3:**
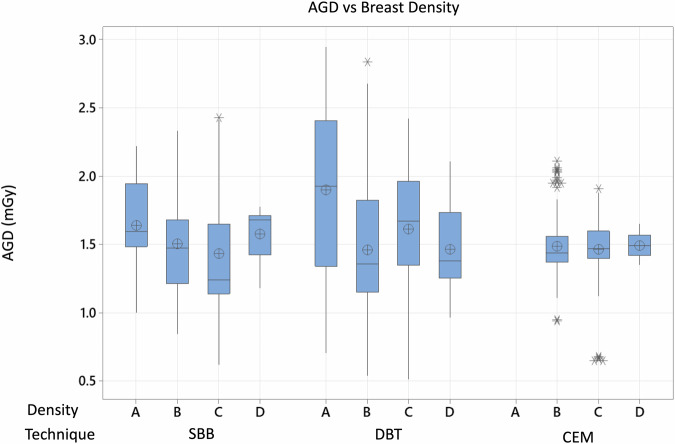
Box plot of AGD values by ACR breast density category and guidance modality. This figure illustrates the spread and central tendency of AGD within each category and modality

Overall, AGD was consistent across ACR breast density categories for all guidance modalities (Table 4 and Fig. 3), except for breast density category A. This category showed an approximate increase of 0.30 mGy in AGD (*p* < 0.001), an observation that correlates with a higher mean CBT for this group, as detailed in Table 1.

### Per-procedure AGD estimation

Utilising AGD data from Table 2 and adhering to our institution’s biopsy protocols (see Supplementary Fig. 1), we estimated the average AGD for each procedure type. For conventional SBB and CEM-guided biopsies, which typically require a minimum of eight images, the estimated AGDs per procedure were approximately 11.92 mGy and 11.84 mGy, respectively. By comparison, DBT-guided biopsies, needing fewer images at an average of 4 per procedure, yielded a lower estimated AGD per procedure, approximately 6.2 mGy.

### AGD repartition in CEM-guided biopsy

**Fig. 4:**
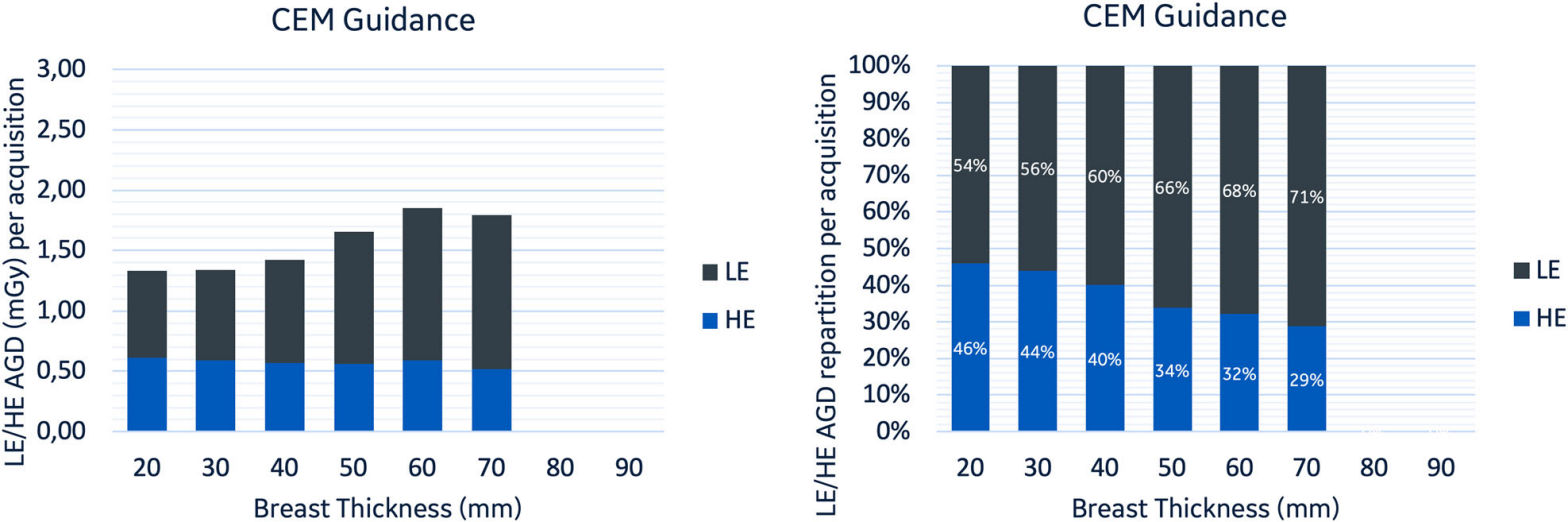
AGD distribution between LE and HE images for CEM-guided biopsies. The left graph displays AGD for LE and HE exposures across breast thickness intervals. The right graph shows the percentage contribution of LE and HE to total AGD. Breast thickness is categorized by ± 5 mm intervals around the central measurement (e.g. 20 mm includes 15–25 mm)

In the subgroup of CEM-guided procedures, the contributions of LE and HE acquisitions to the radiation dose were evaluated. On average, a higher percentage of AGD values is assigned to LE images compared to HE images across all CBT categories (Fig. 4). The proportion of the total dose attributed to LE images varies from 54% to 71%, while the remaining 29% to 46% is associated with HE images.

## Discussion

Mammography-guided biopsies, traditionally reliant on stereotactic methods, have evolved significantly with the introduction of DBT and CEM. CEM has expanded from an MRI alternative to a promising biopsy guidance tool [26], effectively targeting lesions only visible on RC images [2, 3, 27, 28]. Studies show that up to 40% of such lesions are malignant [2, 28, 29], underlining CEM’s essential role in identifying potentially cancerous abnormalities. Despite these technological advances, comprehensive comparisons of AGD across different biopsy techniques are still lacking.

This retrospective study assessed the AGD per acquisition across three mammographic-guided biopsy techniques: SBB, DBT, and CEM. To mitigate variations in the number of acquisitions and procedures, our analysis focused on per-acquisition data rather than entire procedures due to differences in equipment performance and operator learning curves. Additionally, instances of multiple biopsies in a single session increased image counts, deviating from standard procedural workflows. While overall mean AGD per acquisition values were comparable, detailed analysis revealed that AGD for DBT was higher compared to SBB and CEM, particularly when not adjusted for CBT differences. Importantly, all measured AGDs per acquisition remained below the 3 mGy limit set by the Mammography Quality Standards Act [30].

AGD consistently increased with CBT across all modalities. To address potential biases from varying CBT distributions, we stratified AGD per acquisition by CBT levels. At lower CBT levels (20 mm), CEM biopsies showed higher mean AGD than SBB and DBT, but this difference diminished with increased CBT, showing lower AGD for CEM at thicknesses above 50 mm. This consistent dosing profile within CEM across varying thicknesses can be attributed to its AOP settings. Unlike SBB and DBT, which use ‘STD +‘ mode to enhance image quality at a slightly increased dose, CEM operates in ‘STD’ mode, which requires less dose adjustment for thicker breast tissue. These mode differences likely support the specific AGD trends seen in CEM, suggesting a technical basis for its differential dose responses. AGD was relatively consistent across different ACR breast density categories, irrespective of the guidance modality used, except for a slight increase in category A. This increase could be attributed to the higher mean CBT in this category, necessitating greater radiation doses for effective imaging.

The retrospective nature of this study also constrained a comprehensive per-procedure analysis. This focus on per-acquisition data allowed us to standardize comparisons across modalities, considering procedural complexities. While the estimated AGDs per procedure for SBB and CEM were similar, reflecting comparable acquisition counts, DBT-guided biopsies showed lower AGD but lacked detailed data on lesion enhancement—a key factor that CEM guidance confronts, potentially enhancing biopsy accuracy. Our findings, though specific to the equipment used, enrich the ongoing discourse on AGD in CEM-guided biopsies. Our study estimated the procedural AGD for CEM at approximately 11.84 mGy, which is lower than the findings reported by Tang et al [19] at 14.3 mGy and Sammara et al [20] at 14.8 mGy. While these comparisons provide important benchmarks, the small sample size in Tang’s study [19] and the lack of detailed methodology in both points out the need for cautious interpretation. Our research offers a comprehensive analysis of AGD per acquisition and estimated per procedure, enabling direct comparisons with SBB and DBT guidance. Nevertheless, actual AGD values in clinical practice may vary, reflecting the complexity of real-world scenarios.

Furthermore, the increase in AGD for CEM-guided image acquisition was mostly due to an increase in LE dose, aligning with findings by Genaro et al [14] in routine CEM scenarios. This nuanced understanding of AGD variations reinforces the importance of considering the specific contributions of LE doses in CEM’s radiation profile.

Our study underscores the potential of CEM for decision-making in mammography-guided biopsies. Echoing Amir et al [21], who suggest not limiting DBT biopsies to DBT-specific findings, the application of CEM guidance could broaden to encompass more indications, thus enabling more precise targeting and richer morpho-functional insights. Demonstrating that CEM guidance does not lead to higher radiation doses is vital for its wider implementation, highlighting the need for additional, and extensive prospective studies.

This study provides important insights but is limited by its retrospective design and single-centre scope, using specific equipment. Not all consecutive cases were included due to incomplete datasets. AGD calculations, crucial for assessing radiation dose, are based on ideal conditions not always met in biopsy scenarios, hence the results are indicative rather than definitive. Moreover, relying solely on AGD to assess radiation risk might be inadequate, as it does not account for other influential factors such as glandular tissue volume exposed to radiation and varying patient characteristics, including age. Furthermore, our analysis excludes AGD data for breast density category A in CEM-guided biopsies, as this category was not represented due to the absence of RC-only findings in less dense breasts. This limitation might have influenced our overall AGD results, reinforcing the need for further studies to include all breast densities to ensure comprehensive radiation safety evaluations. Future research should utilise a prospective design, expand across multiple centres, include all densities and ensure comprehensive data collection, and consider additional metrics beyond AGD to enhance radiation risk assessment

## Conclusions

In this series, CEM single acquisitions presented AGD values that were on par with those from SBB, and lower than DBT, as demonstrated by the mean AGD metrics obtained using our current imaging equipment. Interestingly, while CEM exhibits higher AGDs in thinner breasts, the increase in AGD with increased CBT is less steep for CEM compared to SBB and DBT, resulting in relatively lower AGD values in thicker breasts. DBT guidance was shown to be more dose-efficient in procedural terms, attributed to fewer image acquisitions. Nevertheless, CEM’s ability to offer enhanced visualization for breast interventions, while adhering to safe radiation parameters, solidifies its potential for expanded clinical application and warrants additional investigative efforts.

## Supplementary information

ELECTRONIC SUPPLEMENTARY MATERIAL

## Abbreviations

ACR: American College of Radiology
AGD: Average glandular dose
AOP: Automatic optimization of parameters
CBT: Compressed breast thickness
CEM: Contrast-enhanced mammography
CI: Confidence interval
DBT: Digital breast tomosynthesis
DM: Digital mammography
HE: High-energy image
LE: Low-energy image
RC: Recombined image
SBB: Stereotactic breast biopsy
STD: Standard AOP mode (GE Healthcare^TM^)
STD +: Standard plus AOP mode (GE Healthcare^TM^)
