# Supplementary material for: Breast radiation dose with contrast-enhanced mammography-guided biopsy: a retrospective comparison with stereotactic and tomosynthesis guidance
Source: Eur Radiol. 2024 Aug 14;35(4):2119–29. doi: 10.1007/s00330-024-10920-3 (PMC11914308; doi:10.1007/s00330-024-10920-3)

Breast radiation dose with contrast-enhanced mammography-guided biopsy: a retrospective comparison with stereotactic and tomosynthesis guidance
ELECTRONIC SUPPLEMENTARY MATERIAL

Eur Radiol (2024) Alcantara R, Azcona J, Pitarch M, et al.

Supplementary Figure 1: Flowchart outlining the procedural steps for estimating AGD in mammography-guided interventions. It compares the steps involved in SBB, DBT, and CEM-guided biopsies, including scout image acquisition, lesion coordinate determination, pre-fire needle alignment, and post-biopsy clip marker placement, as detailed in our institution's practice.

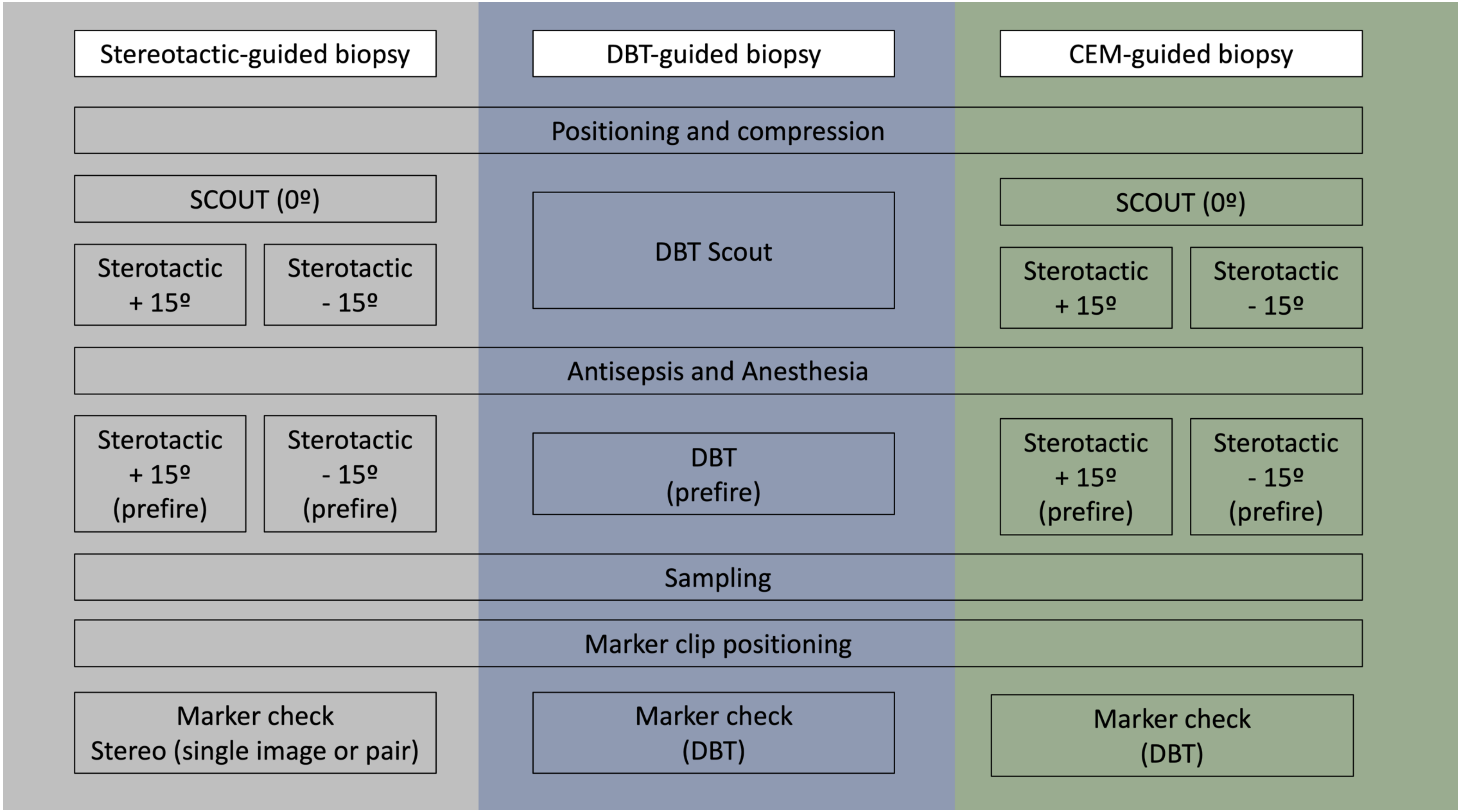

Supplement: Supplementary file 1 — ELECTRONIC SUPPLEMENTARY MATERIAL [file 330_2024_10920_MOESM1_ESM.pdf]
